# Surgical Therapy of Atrial Fibrillation

**DOI:** 10.1155/2012/149503

**Published:** 2012-03-25

**Authors:** Martin Haensig, Ardawan Julian Rastan, David Michael Holzhey, Friedrich-Wilhelm Mohr, Jens Garbade

**Affiliations:** ^1^Department of Cardiac Surgery, Heart Center, University of Leipzig, 04289 Leipzig, Germany; ^2^Department of Cardiac Surgery, Cardiovascular Center, 36199 Rotenburg/Fulda, Germany

## Abstract

Atrial fibrillation (AF) can be found in an increasing number of cardiac surgical patients due to a higher patient's age and comorbidities. Atrial fibrillation is known, however, to be a risk factor for a greater mortality, and one aim of intraoperative AF treatment is to approximate early and long-term survival of AF patients to survival of patients with preoperative sinus rhythm. Today, surgeons are more and more able to perform less complex, that is, minimally invasive cardiac surgical procedures. The evolution of alternative ablation technologies using different energy sources has revolutionized the surgical therapy of atrial fibrillation and allows adding the ablation therapy without adding significant risk. Thus, the surgical treatment of atrial fibrillation in combination with the cardiac surgery procedure allows to improve the postoperative long-term survival and to reduce permanent anticoagulation in these patients. This paper focuses on the variety of incisions, lesion sets, and surgical techniques, as well as energy modalities and results of AF ablation and also summarizes future trends and current devices in use.

## 1. Background

Atrial fibrillation is defined as uncontrolled atrial electrical excitation at a rate of >300 beats per minute. The conduction to the ventricles is irregular and in variable frequencies, therefore resulting in the types of slow (bradycardiac), normofrequent, or fast (tachycardiac) atrial fibrillation (AF). Furthermore, AF can be divided into paroxysmal, persistent, and permanent (accepted) AF [1]. Paroxysmal AF is self-terminating, usually within 48 hours. Although AF paroxysms may continue for up to 7 days, after 48 hours the likelihood of spontaneous conversion is low, and anticoagulation must be considered. Persistent AF is an AF episode which either lasts longer than 7 days or requires termination by cardioversion with drugs or by direct electrical cardioversion. The persistent types of AF are frequently symptomatic and are, depending on the comorbidities, associated with an increased stroke risk. Persistent AF is added by the subtype of long-standing persistent AF (>1y), when it is decided to adopt a rhythm control strategy. Permanent AF is when the presence of the arrhythmia is accepted and a rhythm control is no longer pursued.

Surgical treatment of atrial fibrillation should be considered as a stand-alone concept when patients do not get free of AF or symptoms despite multiple interventional ablations or when a contraindication for catheter ablation exists [1]. Furthermore, atrial fibrillation as a comorbidity can be found in an increasing number of cardiac surgical patients having an indication for coronary artery bypass surgery and valve surgery, respectively [2, 3]. Atrial fibrillation in these patients is known to be a risk factor for mortality over the years after the operation [4]. The surgical treatment of atrial fibrillation in combination with the core cardiac surgical intervention has the aim to improve the postoperative long-term survival of these patients and moreover to reduce the likelihood of permanent anticoagulation. Recently, it has been shown in a randomized trial that surgical ablation is superior to catheter ablation in achieving freedom from left atrial arrhythmias after 12 months of followup [5]. If there are thrombus formations in the left atrial appendage (LAA), or there is an increased risk of stroke, the simultaneous intraoperative resection of the left auricle allows in analogy to the data for the interventional atrial appendage closure a reduction of embolic stroke risk [6]. For surgical treatment of atrial fibrillation today, in contrast to the widely established surgical “cut and sew” technique established in the 1990s by Jimmy Cox, almost always minimal-invasive ablation concepts are applied. Doing so, it is possible to create an effective transmural lesion with low endothelial damage and without adding a significant risk to the cardiac procedure.

## 2. Surgical Techniques for AF Ablation

**Figure 1:**
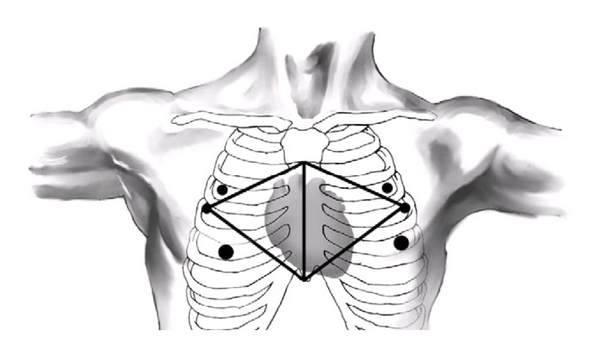
Port access for bilateral minimally invasive AF ablation. AF: atrial fibrillation.

For isolated treatment of atrial fibrillation, there are different surgical approaches available today. Basically, they are now performed endoscopically (Figure 1), whereupon depending on the ablation concept mono- or bilateral thoracoscopic approaches are possible. Because the ablation in this pathology is predominantly performed epicardially, the procedure is usually performed on the beating heart without using the heart-lung machine. This minimal-invasive surgery allows for short operation times of about 2 hours, a rapid recovery, and short hospital stay. Today, the Atricure system (Synergy Ablation Clamp and Coolrail application, Atricure Inc., West Chester, OH, USA) and the Estech device (Cobra adhere XL, San Ramon, CA, USA) are the most commonly used devices for the endoscopic and video-assisted approach (see below). Alternatively, an endocardial approach is possible by using cardiopulmonary bypass connected through the groin vessels and cardioplegic arrest through a right atrial minithoracotomy.

The surgical lesion line concept is based on the modern knowledge of the origin of AF triggers at the connection of the pulmonary veins to the left atrium and the modified concepts of trigger elimination and substrate modification. Based on this, the key element of any ablation is a trigger elimination by circular pulmonary vein isolation [7]. This seems to be sufficient for paroxysmal AF. The lesion line concept has to be expanded, however, to a substrate modification in more complex cases [8]. This is done by a complete isolation of the entire posterior atrial sleeve [9], also known as a box lesion and an ablation of the left atrial isthmus to the posterior mitral valve ring [10]. An additional isolation of the right atrium, which then mimics the complete lines of the early CoxMaze III operation, can also be indicated in selected patients and is called CoxMaze IV procedure [11].

For AF ablation performed concomitantly to cardiac surgical procedures, devices with different energy sources are used. Besides the bipolar radiofrequency energy, cryoapplication by means of argon or nitrous oxide and high-intensity focused ultrasound is used [12, 13]. Depending on the indicated atrial ablation concept the devices offer different advantages and will be used according to the intended lesion concept. The epicardial ablation can be performed on the beating heart. Therefore, epicardial ablation systems will be preferred in patients with simultaneous coronary bypass surgery or aortic valve replacement, whereas endocardial ablation devices are indicated predominantly during surgery for the mitral valve, where the left atrium will be opened in any case during the procedure. Thus, it is possible to select the optimal ablation device according to the appropriate lesion concept and the concomitant cardiac surgical procedure for an individual patient.

## 3. Current Trends and Devices

The used ablation energy source and surgical approach depend on the underlying type of AF and the applied concomitant surgical procedure. Today, most AF surgical procedures are performed as an additional treatment during otherwise indicated cardiac surgery. Conceptionally, an endocardial approach is mostly used when opening of the left atrium is part of the surgical procedure (i.e., mitral valve surgery in most of the cases). In these patients cryoenergy is the energy source of choice. In all other cases an epicardial approach is favourable to reduce cross-clamping time and morbidity due to blood loss and prolonged cardiopulmonary bypass time. For the epicardial approach, a pulmonary vein isolation (PVI) is indicated in paroxysmal AF by using the different ablation clamps mentioned above. In more complex AF patients, however, epicardial box lesions and even epicardial left isthmus ablation are possible by radiofrequency energy or high-intensity focused ultrasound.

## 4. Radiofrequency Energy

AtriCure (West Chester, OH, USA) markets the Isolator Synergy Ablation Clamp that uses bipolar energy. The distal end consists of two electrodes separated by an insulation. Because the Synergy Ablation Clamp focuses the energy directly into the contacted tissue, it can perform with higher efficiency than can unipolar configurations while limiting damage to tissue that is in close proximity to the electrodes.

Medtronic (Minneapolis, MN, USA) first developed the Cardioblate pen. It is a pen-like device used to make point-by-point ablations by dragging it across tissue to make a linear lesion. Medtronic also markets three bipolar clamp devices, all with irrigated flexible jaws and an articulating head. The Cardioblate BP has 5 cm-long electrodes. Cardioblate BP2 has a flexible neck and longer, 7 cm, electrodes. A low-profile device was developed, Cardioblate LP, featuring a new sleek design.

In addition, Estech (San Ramon, CA, USA) offers two surgical probes: the Cobra Surgical Probe and the Cobra Cooled Surgical Probe. Both are segmented, flexible devices with multiple electrodes. The cooled probe has internal saline cooling. The Cobra Revolution Bipolar Clamp (Figure 3) automatically adjusts power output to achieve and maintain a safe and effective target temperature of >50°C. The system adjusts electrode output to accommodate varying tissue depth. To maintain probe position during beating-heart applications, the Cobra Adhere provides suction stabilization to the probe device, whereas the Cobra Adhere XL does so for a minimally invasive approach.

**Figure 2:**
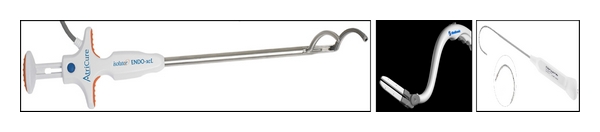
Modern radiofrequency energy ablation devices, Atricure Isolator Synergy^TM^ Ablation Clamp (left), Medtronic Cardioblate^TM^ BP2 (middle), and Estech Cobra^TM^ Surgical Probe (right).

**Figure 3:**
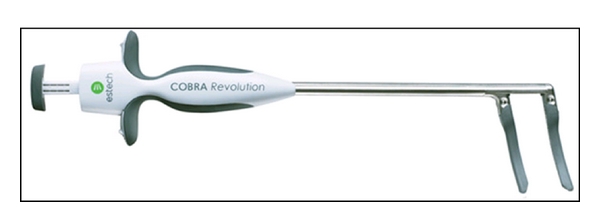
Cobra^TM^ Revolution Bipolar Clamp (Estech, San Ramon, CA, USA).

Radiofrequency energy has been extensively used since many years in the electrophysiological laboratory and operating theatre (Figure 2) [14]. Alternating current generates electromagnetic energy of frequencies between 350 and 1000 kHz. Advantages are the precise and transmural lesions by measurement of tissue resistance, the avoidance of collateral damage, and its easy usage during epicardial application [15, 16]. Besides a potential risk of thrombogenicity of ablation lesion lines, a full epicardial/endocardial box lesion can be demanding [17].

## 5. Cryoablation

**Figure 4:**
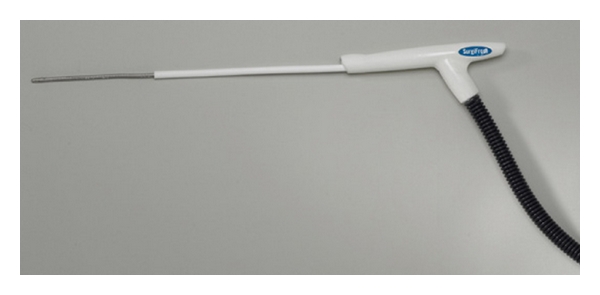
Argon-based ATS CryoMaze^TM^ surgical ablation system.

The argon-based ATS CryoMaze Surgical Ablation System (former SurgiFrost System, CryoCath Technologies, Inc., Figure 4) offers a two-in-one convertible heart ablation system with a clamp (FrostByte) and two surgical probes (CryoMaze 7 cm and 10 cm). The system is versatile enough to be used endocardially and epicardially on the beating or arrested heart [18].

**Figure 5:**
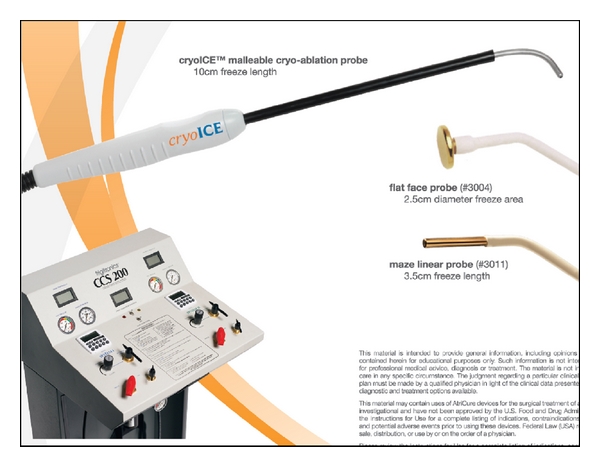
N_2_O-based CryoICE^TM^ Probe (Atricure, Inc., West Chester, OH, USA).

**Figure 6:**
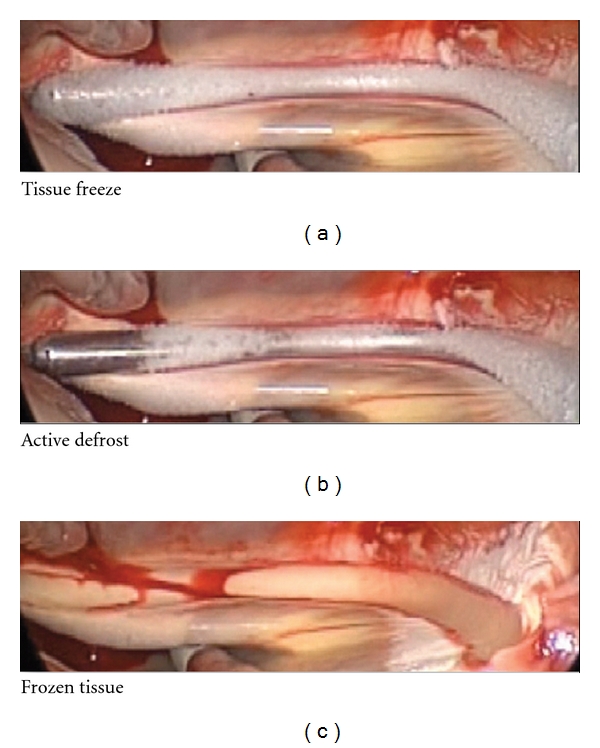
Freeze cycle of the CryoICE^TM^ Probe.

More recently, Atricure introduced a novel N_2_O-based CryoICE Probe to the market (Figures 5 and 6). The device has a 10 cm malleable aluminum probe with a retractable handle. N_2_O has a higher heat absorption capacity than argon gas that requires lower gas pressures to provide sufficient cooling (max. −55 to −60°C) and no thermal insulation. An active feedback feature at the probe/tissue interface allows maintaining temperatures along the probe length more consistently.

Cryoablation is, next to radiofrequency ablation (RF), the most frequently employed alternative method to create linear, continuous, and transmural lesions. Application of cold by fast-expanding N_2_O (Boyle's law) or by Argon or Helium (max. −160°C) lead to a three-step tissue injury (freeze and thaw, inflammation, and fibrosis). Advantages are the benefit of preserving the fibrous skeleton of the heart, the improved safety margin for ablation near the coronary arteries or atrioventricular node, and its low incidence of thrombus formation. Besides the relatively long time necessary per lesion (2 to 5 minutes), a higher recurrence rate compared to radiofrequency energy has been reported [19]. Lesions on the beating heart might be ineffective, and mild esophageal lesions are described [20].

## 6. Microwave

The Flex 4 and Flex 10 ablation probes are the only intraoperative, single-use devices designed to apply microwave energy [21, 22]. Both were previously marketed by Guidant Inc. (Boston Scientific, Natick, MA, USA). The Flex 4 has a flexible 4 cm-long sheath to ablate in increments of 2 cm; the Flex 10 is 20 cm long and also ablates in 2-cm increments. The ablations are created by independently activating the microwave ablation element at one or more of the corresponding numbered segments, which are selected by moving the sliding ring on the handle.

Microwave energy ablation may offer the advantage of a greater tissue penetration than radiofrequency ablation, increasing the likelihood of a transmural lesion. In addition, microwave heating does not cause endocardial surface charring, which may reduce the risk of thromboembolism [12]. However, these theoretical advantages of microwave energy could not avoid the decline of both surgical devices.

## 7. Laser Energy

MedicalCV, Inc. (Inver Grove Heights, MN, USA) markets the AtriLaze Surgical Ablation System, which uses an 810 nm wave length, 25 W diode laser. A focused, narrow beam allows for 3 mm lesions to minimize collateral damage [23].

By laser energy, precise, focused, and well-demarcated lesions can be achieved, but a nearly perpendicular delivery angle is required for efficient energy delivery, and an increased risk for atrial thrombus formation has been observed [23].

## 8. High-Intensity Focused Ultrasound

**Figure 7:**
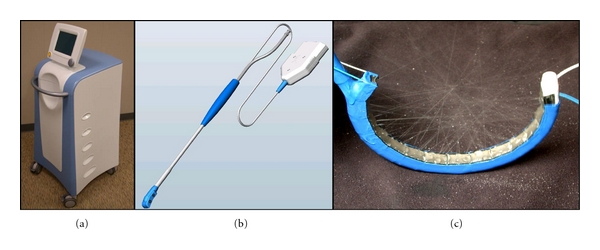
Modern high-intensity focused ultrasound (HIFU) ablation device, Epicor^TM^ System Console (a), Epicor^TM^ UltraWand LP (b) and medical UltraCinch LP ablation device (c). LP: low profile.

The Epicor Ablation System (Figure 7, St. Jude Medical, Inc., St. Paul, MN, USA) is intended specifically for epicardial, off-pump use and is designed to create a classic box lesion in a single step [24]. By using the PAS system proper sizing of the Epicor Medical UltraCinch LP device can be assured. Seven different sizes allow for appropriate device selection. The UltraWand^TM^ LP is a handheld device designed to create linear lesions to complement the Epicor^TM^ UltraCinch by aiming an epicardial left atrial isthmus ablation.

Ultrasound allows for the development of heat by oscillation of aqueous tissue. Advantages are the tissue penetration independently of the surrounding fatty tissue (within less than 2 sec), effective box lesions without electrical gaps, and the adaption to tissue thickness [25]. The endothelium is relatively spared; hence, the risk of thrombus formation is considered to be comparably low.

## 9. Results of AF Ablation

**Figure 8:**
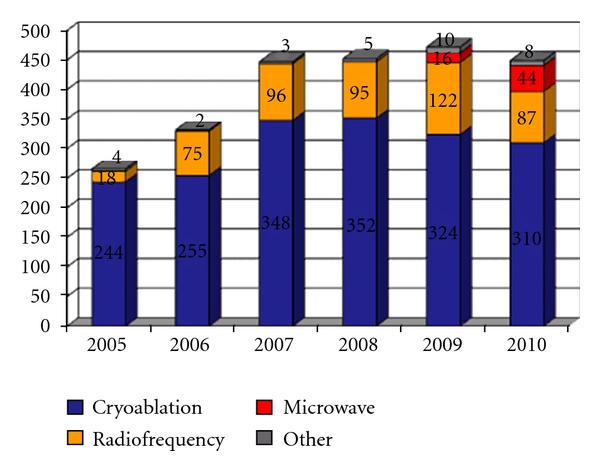
AF ablation at the Heart Center Leipzig 2005–2010, *n* = 2.418.

At the Leipzig Heart Center between the years 2005 are 2010 in total more than 2,400 ablations for the treatment of atrial fibrillation have been carried out (Figure 8). Due to the new minimal-invasive procedures we did not find any serious ablation-associated complication. As the success of therapy depends in particular on the etiology of atrial fibrillation, in recent years an etiology-based analysis of the world's largest ablation collective has been performed. For isolated atrial fibrillation a success rate of more than 90% after one year can be expected, although this was determined in paroxysmal AF patients who underwent no preoperative percutaneous ablation. Due to the current patient selection for percutaneous treatment failures a slightly lower overall success rate may be assumed.

For 262 patients with an ablation therapy in conjunction with coronary artery bypass surgery in our institution, the success rate of ischemic AF etiology was the most difficult one to assess in comparison to idiopathic or valvular AF. The rate of sinus rhythm in this group was 75% after one year in paroxysmal AF, but only at 55% in the persistent type of atrial fibrillation. Interestingly, these patients did not show a higher success rate when a more complex ablation concept including a substrate modification was chosen. As a consequence we now consider these patients for an off-pump bypass surgery approach with an epicardial ablation concept using a pulmonary vein isolation in paroxysmal AF and a full box lesion in persistent AF types. To date, neither the success rate nor the survival rates of surgical ablation in the different etiological groups (lone, valvular and ischemic AF) can be definitively recommended on the basis of comparative trials, even if some loose estimates exist [26, 27].

**Figure 9:**
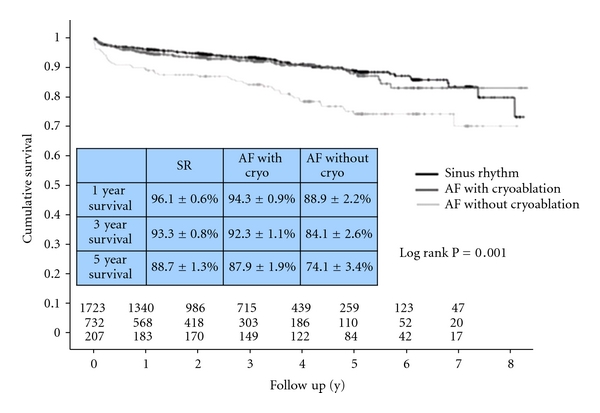
Cumulative survival of patients with sinus rhythm and atrial fibrillation with and without cryoablation after mitral value surgery.

A comparable study of patients who received AF ablation by cryoapplication during minimal-invasive mitral valve surgery through a right lateral minithoracotomy showed comparably better results. The lesion concept in these patients corresponds to pulmonary vein isolation with additional isolation of the posterior atrial sleeve and a lesion to the posterior mitral valve annulus in order to modify the atrial substrate. In these patients, freedom from AF could be reached in more than 70% [28]. Of utmost importance was that for these patients also a significant improvement of survival was found, which was closed to that of patients with sinus rhythm and which was significantly better than that of comparable patients that did not receive intraoperative AF ablation. Thus, for the first time it could be shown in more than 700 mitral AF patients that ablation of preexisting atrial fibrillation was associated with a survival advantage compared with no ablation (Figure 9). Therefore, the ablation strategy today represents a fundamental part during minimally invasive mitral valve surgery in patients with preoperative AF.

## 10. Occlusion of the Left Atrial Appendage

### 10.1. Background

Patients with atrial fibrillation are prone to an at least 5- to 7-fold increased risk for developing a thromboembolic stroke event [29]. Based on well-defined risk scores (CHADS_2_, CHA_2_DS_2_-Vasc), almost all cardiac surgical patients with AF require long-term anticoagulation. Due to the alternative interventional occlusion of the left atrial appendage as the main source of thromboembolic formations a comparable event rate compared to patients with permanent anticoagulation could be demonstrated [30]. Therefore, in addition to AF ablation the prophylactic occlusion of the left atrial appendage represents a treatment option for AF patients. This is even more important in persistent AF where, as shown above, a success rate was limited to 55–75% only, and especially in the first months after AF treatment numerous short episodes of AF occur. Thus, besides ablation, the occlusion of the atrial appendage probably allows AF patients with a CHADS_2_ Score ≥ 1 the greatest protection against stroke, in particular after discontinuation of oral anticoagulation [1].

### 10.2. Surgical Procedure of Atrial Appendage Occlusion

**Figure 10:**
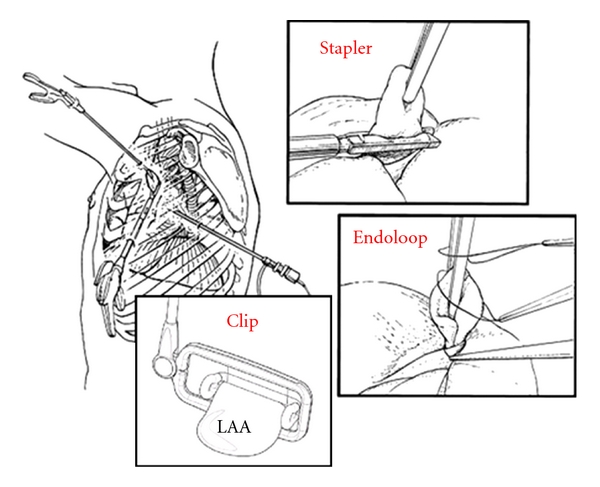
Surgical procedure of atrial appendage occlusion by the use of endoloop systems, staplers, or clips.

For the surgical occlusion of the atrial appendage several options are available. These include an endocardial occlusion of the orifice of the left atrial appendage to the left atrium, the resection/amputation of the appendage with following double-row stitches, or epicardial occlusion at its base while leaving the appendage in situ. The latter is possible by surgical purse-string sutures, but also, the use of endoloop systems, staplers, and more recently clips (Figure 10). The use of endoloops, staplers, or clips offers advantages in particular within the scope of videoscopic and minimal-invasive ablation procedures. Due to the different sizes and variable anatomy of the left atrial appendage, different sizes of staplers and clips are available. For all procedures it is important to look for a gentle approach, because the left atrial appendage is sometimes very thin, and injuries can occur easily.

### 10.3. Gillinov-Cosgrove LAA Exclusion System

The AtriClip (Atricure, Inc., West Chester, OH, USA) is developed for the occlusion of the left atrial appendage, under direct visualization, in conjunction with other open cardiac surgical procedures. The AtriClip is available in various sizes, can easily be repositioned prior to suture release, and closes as tissue is compressed. While the orifice is permanently sealed with a low compression force between 2 to 8 psi, the tissue is fully absorbed.

### 10.4. Results of Atrial Appendage Occlusion

Based on several observational studies a prophylactic occlusion of the left atrial appendage seems suitable to avoid subsequent strokes in the scope of cardiac surgery [31]. In addition, it could be shown based on a study of 137 patients that in comparison of excision/resection against the exclusion by sutures or staplers, the excision represented the more effective therapy with respect to the presence of residual niches at the base of the atrial appendage [32]. The extent to which this can be transferred to today's exclusion systems is unknown, but current data, however, suggest a better success with modern exclusion systems [6]. In either case the optimal position and the occlusion has to be controlled by intraoperative transesophageal echocardiography.

### 10.5. Summary

The introduction of new AF ablation technologies has revolutionized the surgical therapy of atrial fibrillation. Atrial fibrillation can be found as a comorbidity in an increasing number of cardiac surgical patients due to increasing patient age having an indication for coronary artery bypass or valve surgery.

While modern surgical ablation concepts are based on the origin of AF triggers and the modified concepts of trigger elimination and substrate modification, new minimal invasive techniques allow for short operation times, rapid recovery, and short hospital stays of a few days. Epicardial ablation systems will be preferred in patients with simultaneous coronary bypass surgery or aortic valve replacement, whereas endocardial ablation devices are indicated predominantly during surgery for the mitral valve, where the left atrium will be opened. In addition, novel devices designed specifically for minimally invasive epicardial exclusion of the left atrial appendage will broaden the range of treatment options available.

Surgical AF ablation is a procedure in evolution with a variety of incisions, lesion sets, and energy modalities in use. To optimise its potential, further studies of their efficacy and safety will guide their future development.
